# CREATING NEW MODELS FOR AGING IN PLACE: DIVERSE COMMUNITIES AND THE NAVIGATION OF LONG-TERM SERVICES AND SUPPORTS

**DOI:** 10.1093/geroni/igz038.843

**Published:** 2019-11-08

**Authors:** Lauren Ring, Michael Liebman, Allen Glicksman, Misha Rodriguez

**Affiliations:** 1 Philadelphia Corporation for Aging, Philadelphia, Pennsylvania, United States; 2 IPQ Analytics, LLC, Kennett Square, Pennsylvania, United States; 3 Asociación Puertorriqueños en Marcha, Philadelphia, Pennsylvania, United States

## Abstract

There is a growing interest among aging services providers to better understand the pathways through which older adults and their caregivers navigate LTSS. Although there have been attempts at modeling this process they are often dependent on the quality of existing data, which can result in models which are incomplete and study samples that homogenize diverse older adult populations. These models face two challenges – 1) the data may not include information about important elements of the LTSS navigation process, and 2) the actions of ethnic/cultural sub-groups may not be captured. This study uses a conceptual method called Social Interaction Modeling (SIM) to examine how older adults in two limited English-speaking communities (Spanish / Mandarin Chinese) navigate the use of LTSS and to evaluate disparities in service access. The findings will help to build a more comprehensive model which looks at service navigation among all older adults in Philadelphia.

